# Supplementary material for: High temperature and humidity in the environment disrupt bile acid metabolism, the gut microbiome, and GLP-1 secretion in mice
Source: Commun Biol. 2024 Apr 17;7:465. doi: 10.1038/s42003-024-06158-w (PMC11024098; doi:10.1038/s42003-024-06158-w)
Supplement: Supplementary file 2 — Supplementary Material [file 42003_2024_6158_MOESM2_ESM.pdf]

Supplementary Materials for

High temperature and humidity in the environment disrupt bile acid metabolism, the gut microbiome, and GLP-1 secretion in mice

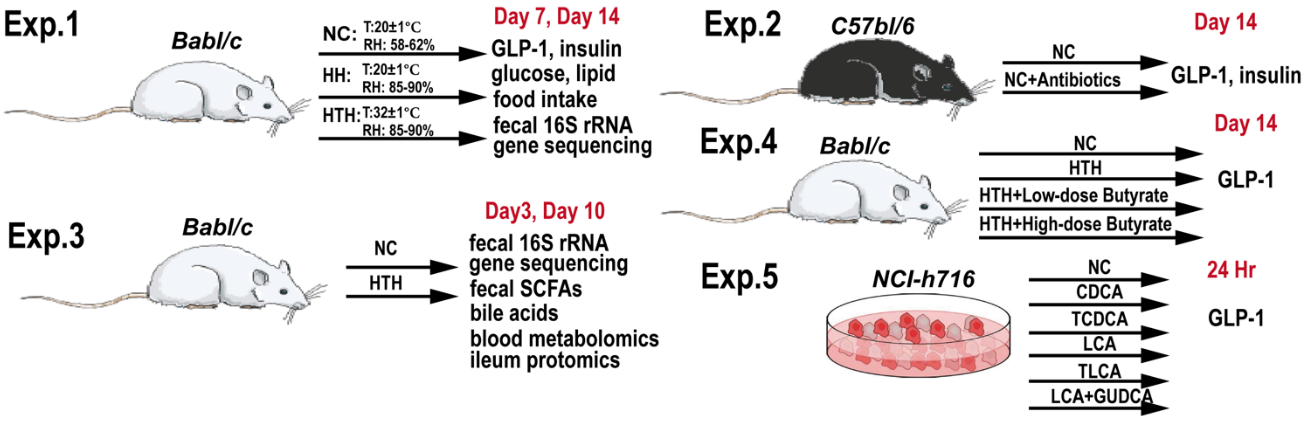

Figure S1. Schematic diagram of the experiment design.

NC: normal control group. HH: high humidity. HTH: high temperature and high humidity.

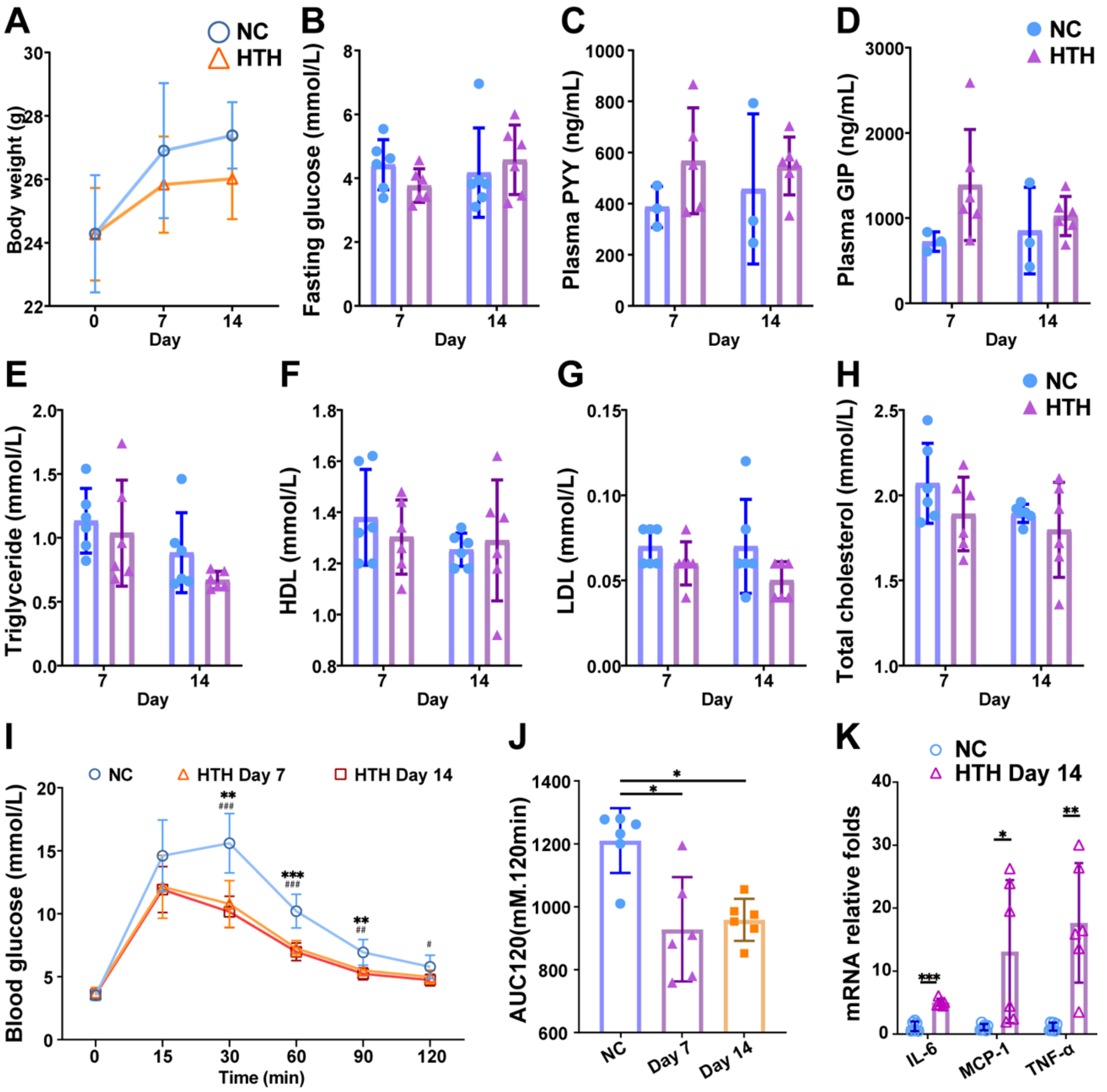

Figure S2. Phenotypic parameters after high-temperature and humidity (HTH) exposure in BABL/c mice. Body weight. (b) Plasma fasting glucose levels. (c) Plasma peptide YY (PYY) levels. (d) Plasma gastric inhibitory polypeptide (GIP) levels. (e) Plasma triglyceride levels. (f) Plasma high-density lipoprotein (HDL) levels. (g) Plasma low-density lipoprotein (LDL) levels. (h) Plasma total cholesterol levels. The difference between HTH and normal control (NC) groups was not significant. (i) and (j)OGTT. (i): NC VS HTH Day 7:$^{**}P< 0.01$, $^{***}P< 0.001$ by one-way ANOVA; NC VS HTH Day 14: $^{\#}P< 0.05$, $^{\#\#}P< 0.01$, $^{\#\#\#}P< 0.001$ by one-way ANOVA; Error bars represent ±s.d. (j): $^{*}P< 0.05$ by Kruskal-Wallis. Error bars represent ±s.d. (k) The mRNA expression of IL-6, MCP-1, TNF-α in colon. n=6 mice for each group. $^{*}P<0.05$, $^{**}P< 0.01$, $^{***}P< 0.001$ by two-tailed Student's t-test. Error bars represent ±s.d.

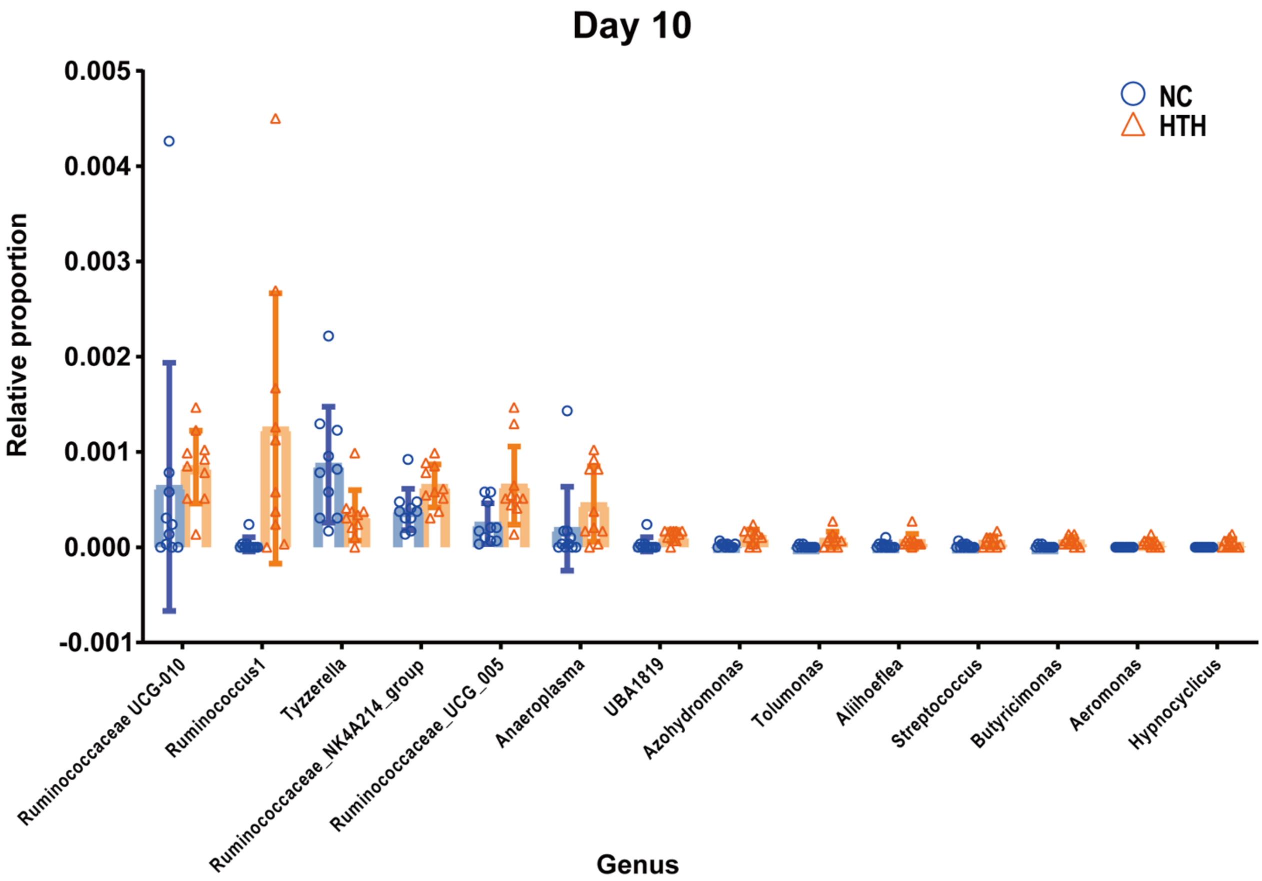

Figure S3. The relative proportion of genus with differential abundance identified at day 10 in HTH treated mice (Exp.3).

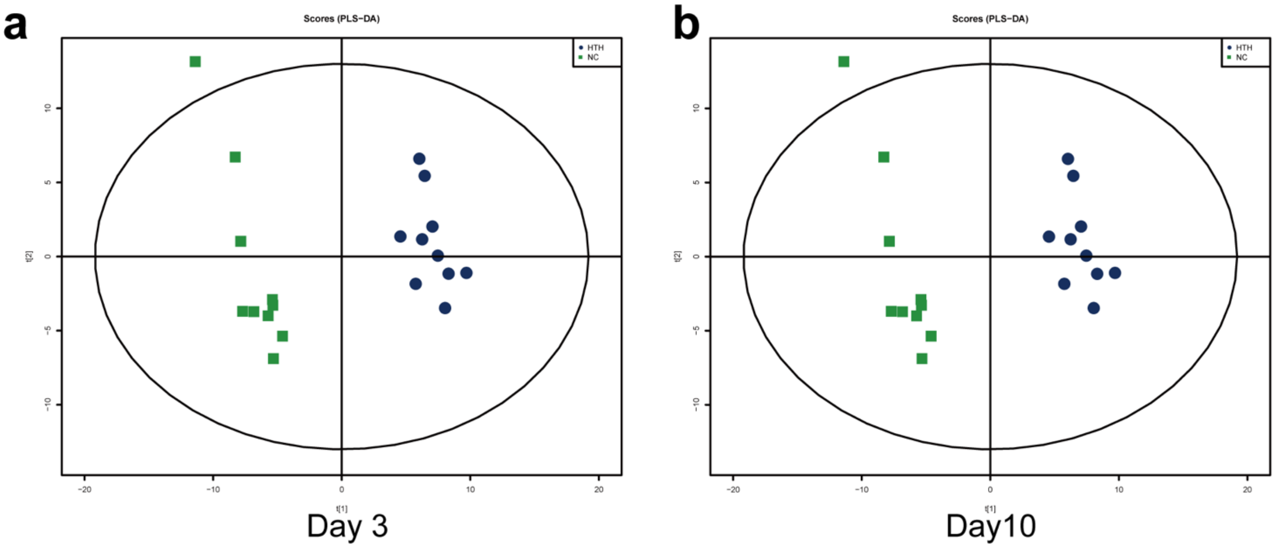

Figure S4. Scatter plots obtained from ultra-high performance liquid chromatography-quadrupole time-of-flight mass spectrometry (UPLC-Q-TOF MS) analysis.

(a) (b) Partial least-squares-discriminant analysis (PLS-DA) plasma samples at day 3 or day 10, respectively.

.

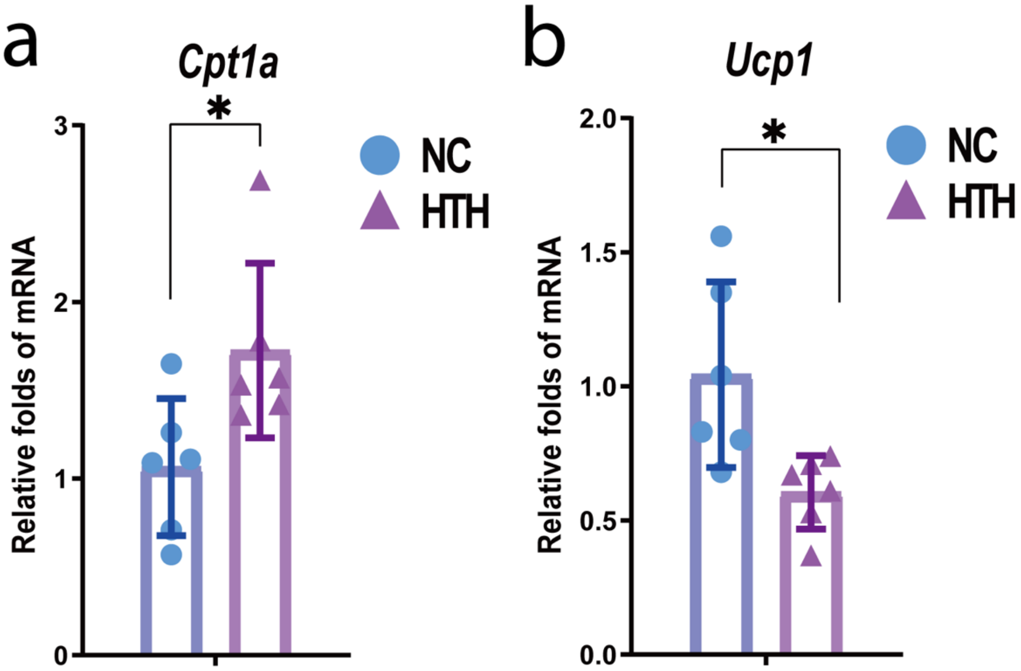

Figure S5. Quantitation of hepatic *Cpt1a* and *UCP1* mRNA levels.

(a)Relative folds of hepatic *Cpt1a* and (b) *Ucp1* mRNA normalized to housekeeping gene *Gapdh* at week 2 after HTH treatment. *n*=6 mice for each group. (a)**P*<0.05 by two-tailed Student's t-test. (b)**P*<0.05 by Mann-Whitney U test. Error bars represent ±s.d.

Supplementary Table 1

Outcome	Exposure	MR method	Number of SNPs	Beta	SE	OR	Association *P*-value	95% confidence interval	Heterogeneity *P*-value
GLP-1	*g_Alistipe*	IInverse-variance weighted	14	1.364	0.552	3.912	0.013	0.283-2.445	>0.05
GLP-1	*g_Alistipe*	MR Egger	14	0.359	2.630	1.431	0.89	-4.795-5.513	>0.05

MR, Mendelian randomization; SNP, single nucleotide polymorphism; Beta, beta coefficient; SE, standard error; OR, Odds Ratio.
