# Supplementary material for: High temperature and humidity in the environment disrupt bile acid metabolism, the gut microbiome, and GLP-1 secretion in mice
Source: Commun Biol. 2024 Apr 17;7:465. doi: 10.1038/s42003-024-06158-w (PMC11024098; doi:10.1038/s42003-024-06158-w)
Supplement: Supplementary file 3 — Description of additional supplementary files [file 42003_2024_6158_MOESM3_ESM.docx]

Description of Additional Supplementary Files

**File name:** Supplementary Data 1

**Description:** The source data behind the graphs in the paper.

**File name:** Supplementary Data 2

**Description:** The source data behind the graphs in the paper.

**File name:** Supplementary Data 3

**Description:** The source data behind the graphs in the paper.
